# Supplementary material for: Conditional chemoconnectomics (cCCTomics) as a strategy for efficient and conditional targeting of chemical transmission
Source: eLife. 2024 Apr 30;12:RP91927. doi: 10.7554/eLife.91927 (PMC11060718; doi:10.7554/eLife.91927)
Supplement: Figure 3—source data 3. [file elife-91927-fig3-data3.zip › Figure 3 source data3 part 2.zip!/Figure 3-Source data 3-part2/1053f3-1-CRISPResso_Report_t6djfp6u.zip!/Nucleotide_percentage_quilt.pdf]

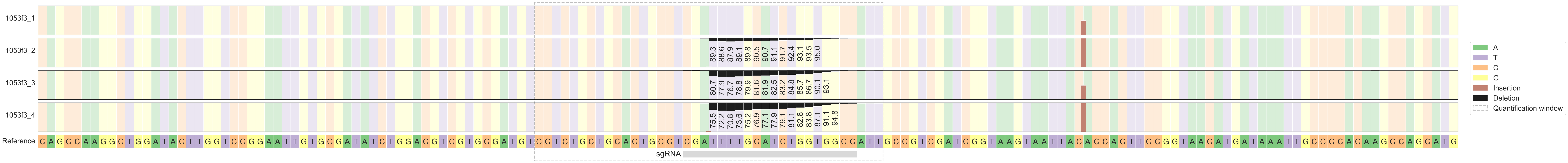

1053f3_1
1053f3_2
89.3 88.6 87.9 89.1 89.8 90.5 90.7 91.1 91.7 92.4 93.1 93.5 95.0
1053f3_3
80.7 77.9 76.7 78.8 79.9 81.6 81.9 82.5 83.2 84.8 85.7 86.7 90.1 93.1
1053f3_4
75.5 72.2 70.8 73.6 75.2 76.9 77.1 77.9 79.1 81.1 82.8 83.8 87.1 91.1 94.8
Reference
CAGCCAAGGCTGGATACTTGGTCCGGAATTGTGCGATATCTGGACGTCGTGCGATGTCCTCTGCTGCACTGCCTCGATTTTGCATCTGGTGGCCATTGCCGTCGATCGGTAAGTAATTACACCACTTCCGGTAACATGATAAATTGCCCCACAAGCCAGCATG
sgRNA
A
T
C
G
Insertion
Deletion
Quantification window
